# Transmembrane Helices Tilt, Bend, Slide, Torque, and Unwind between Functional States of Rhodopsin

**DOI:** 10.1038/srep34129

**Published:** 2016-09-23

**Authors:** Zhong Ren, Peter X. Ren, Rohith Balusu, Xiaojing Yang

**Affiliations:** 1 Department of Chemistry, University of Illinois at Chicago, Chicago, IL 60607, USA; 2 Renz Research, Inc., Westmont, IL 60559, USA; 3 Hinsdale Central High School, Hinsdale, IL 60521, USA; 4 Department of Ophthalmology and Vision Sciences, University of Illinois at Chicago, Chicago, IL 60607, USA

## Abstract

The seven-helical bundle of rhodopsin and other G-protein coupled receptors undergoes structural rearrangements as the transmembrane receptor protein is activated. These structural changes are known to involve tilting and bending of various transmembrane helices. However, the cause and effect relationship among structural events leading to a cytoplasmic crevasse for G-protein binding is less well defined. Here we present a mathematical model of the protein helix and a simple procedure to determine multiple parameters that offer precise depiction of a helical conformation. A comprehensive survey of bovine rhodopsin structures shows that the helical rearrangements during the activation of rhodopsin involve a variety of angular and linear motions such as torsion, unwinding, and sliding in addition to the previously reported tilting and bending. These hitherto undefined motion components unify the results obtained from different experimental approaches, and demonstrate conformational similarity between the active opsin structure and the photoactivated structures *in crystallo* near the retinal anchor despite their marked differences.

The helix is the most common secondary structure found in the transmembrane (TM) segments of membrane proteins. TM helices often undergo concerted motions when a membrane protein is at work, whether it transduces signals across the membrane or facilitates opening and closing of an ion channel. Tilting and bending of TM helices have been observed when protein structures captured in different functional states are compared. A helix as a rigid body may tilt its angle with respect to other parts of the structure or deform as a flexible rod between the straight and bent shapes. In this work, we demonstrate that TM helices exhibit far more complex motions than simple tilting and bending (Supplementary text and Fig. S4). We present an in-depth survey and analysis of the seven TM helices (TM1-7) from bovine rhodopsin, a well-studied G-protein coupled receptor (GPCR) from the rod photoreceptor cells in retina^1^. The dark state of rhodopsin is armed with an 11-*cis* retinal covalently anchored to Lys296 in TM7 as an inverse agonist via a protonated Schiff base. Absorption of a photon triggers isomerization of the retinal chromophore from an 11-*cis* to an all-*trans* conformation. Subsequent deprotonation of the Schiff base eventually leads to release of the retinal and formation of the apoprotein opsin in the active form.

In this work, we reveal that some TM segments in rhodopsin exhibit sliding in the longitudinal direction normal to the plane of the membrane like pistons. Some TM segments undergo significant torsional motions, that is, a rigid body rotation about the helical axis. Two adjacent helices may torque in opposite directions like two meshing gears transmitting motions from one to the other. Furthermore, TM helices may wind and unwind like torsion springs. A helix can also transform from an α conformation to 3/10 or π conformations. Interestingly, two segments of the same helix could be wound differentially when both stay in the α conformation. Most importantly, we find a strong correlation between these movements of helices and the functional states of rhodopsin and opsin. These findings lead us to hypothesize that the TM segments of rhodopsin, and presumably other GPCRs, act as electromechanical parts that interconvert between an inactive but armed state in dark and the active state characterized by the outward tilting of TM6, as the G-protein binding crevasse opens. The structural events triggered by photoisomerization of retinal run through a sequence of transient conformations coupled with various changes in the TM helices identified here, and eventually releases the strains in the helical bundle^2^.

The prokaryotic counterparts to GPCRs that transmit signals across the cell membrane often adopt the dimeric four-helical bundle HAMP domain commonly occurring in *h*istidine kinases, *a*denylyl cyclases, *m*ethyl-accepting chemotaxis proteins, and *p*hosphatases^3^. It has been extensively studied that helices in such a TM bundle undergo torsional motions or axial rotations^4^,^5^,^6^. Therefore, angular motions of helices such as torsion, overwinding and unwinding play a structural role in signal transduction due to the mechanical property of protein helices.

## Results

**Figure 1:**
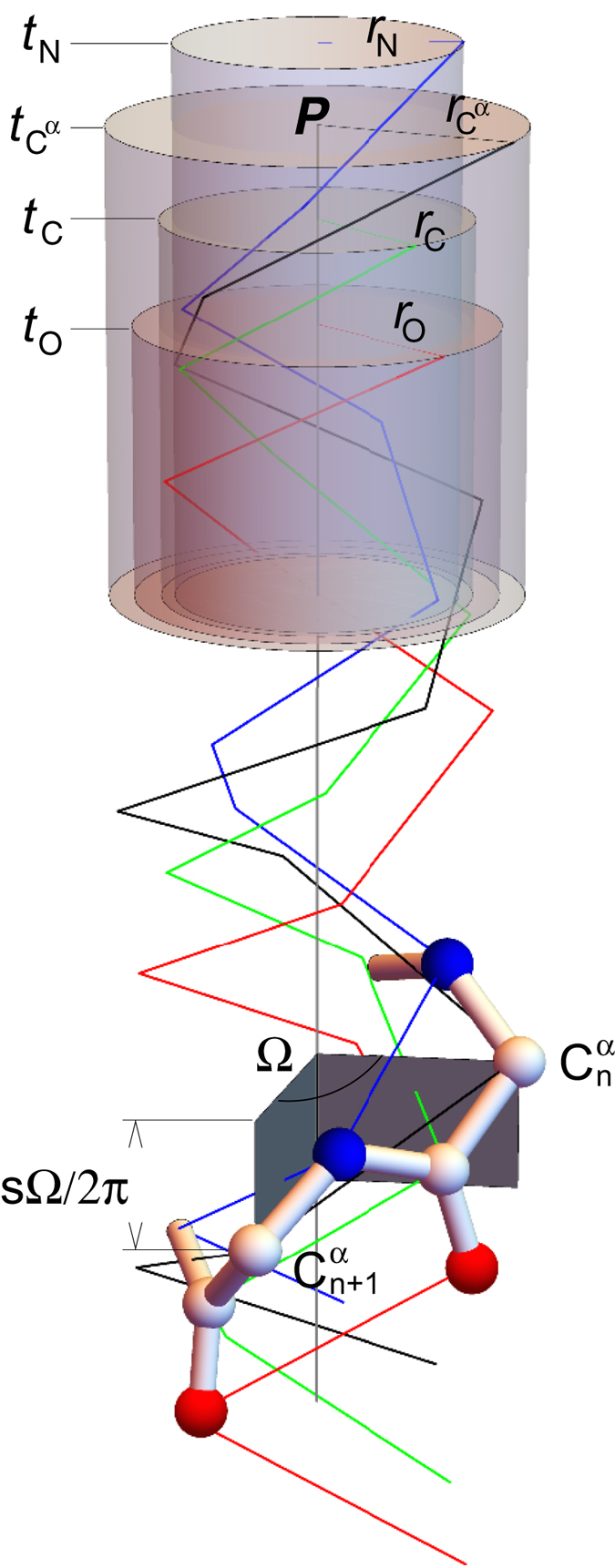
Parameterization of protein helix. A protein helix is modeled as a combination of four sub-helices in blue (N), black (C^α^), green (C), and red (O) for different types of atoms in the main chain (Eq. 1 and Methods). C, N, and O atoms are in white, blue, and red, respectively. These sub-helices share the same axis (vertical gray line), but are of different radii (*r*N, *r*C^α^, *r*C, and *r*O). Four semitransparent cylinders of different thicknesses enclose the sub-helices. The bottom halves of the cylinders are omitted for clarity. The top surfaces of the cylinders are offset from one another because each sub-helix translates along the axis (*t*_N_, *t*_C_^α^, *t*_C_, and *t*_O_) with respect to one another. The sub-helices are also angularly offset by different phases (*φ*_N_, *φ*_C_^α^, *φ*_C_, and *φ*_O_) shown as the directions of radii on the top surfaces of the cylinders. The angular turn per residue *Ω* is the angle between two gray planes that pass the common axis and two consecutive atoms of the same type. The width of the gray planes along the axis equals to *sΩ*/2*π*, where *s* is the helical pitch shared by four sub-helices.

We have formulated a multi-parameter mathematical expression to depict a protein helix (Eq. 1), including its location, orientation, and shape (Fig. 1 and Methods). Least-square fitting of this formula to the atomic coordinates of bovine rhodopsin in Protein Data Bank (PDB) produces a set of geometric parameters that numerically define the conformation of each helix segment in a rhodopsin structure. In addition, the geometric relationship between two helices can be quantitatively analyzed to examine possible conformational interplays. The currently available PDB entries of rhodopsin can be divided into three functional groups (see Methods for detail). The first group includes the ground state rhodopsin structures as well as early intermediates such as batho and lumirhodopsins. The second group features the elongated TM5 and TM6 at their cytoplasmic ends compared to the first group. The opsin and metarhodopsin II structures that feature the characteristic tilt of TM6 constitute the last group. The functional relevance of the changes in helical conformation and interhelix motions are assessed by correlations between these quantitative measures and the functional states of rhodopsin and opsin. This analysis based on helix parameterization reveals abundant and consistent conformational changes, which allow us to infer flows of force, motion, and energy through various parts of the helical bundle. We aim to identify cause-effect relationship among these structural changes that provides insights into the operating mechanism of this photoreceptor protein.

In brief, our joint analysis of helical parameters from a collection of rhodopsin structures demonstrates that the retraction of Try223 from an exposed position in rhodopsin into the molecular interior in opsin, a key structural event discovered in the active state opsin structure^7^,^8^, is caused by helical unwinding of TM5. We also uncover that the rhodopsin structures, which were photoactivated *in crystallo*^9^,^10^, conceal an important structural feature characteristic to those of opsin near the retinal anchor, although the overall structure largely retains the helical bundle arrangement of rhodopsin. Furthermore, our analysis confirms a torsional component of TM6 motion upon photoactivation that has been previously observed by electron paramagnetic resonance (EPR) spectroscopy^11^.

### Winding and unwinding

**Figure 2:**
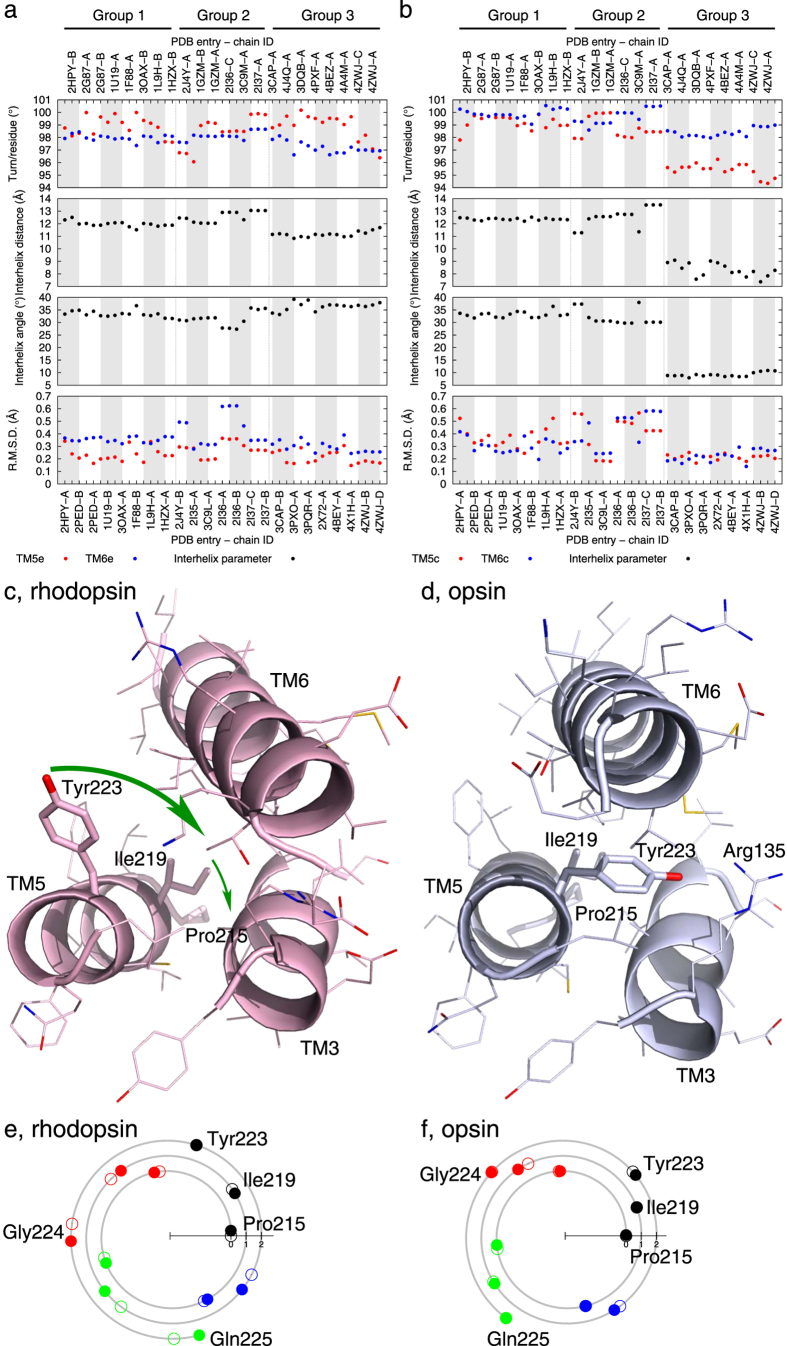
Helix unwinding of TM5 and TM6. The parameter of angular turn per residue has been refined for both the extracellular (**a**) and cytoplasmic (**b**) segments of TM5 (red) and TM6 (blue). See also Movie S1 for the unwinding of TM6 in action. The distance and angle between the helical segments on the extracellular and cytoplasmic sides are also plotted in black in (**a, b**), respectively. See Methods for details of parameter fitting and plotting. TM3, 5, and 6 are viewed from the cytoplasmic side in rhodopsin (**c**) and opsin (**d**). N, O, and S atoms are in blue, red, and gold, respectively. The large and small green arrows indicate the movements of Try223 and Ile219. The spiral diagrams show the experimentally observed and least-squares fitted phases in dot and circle, respectively, for each residue in the cytoplasmic segment of TM5 in rhodopsin (**e**) and opsin (**f**). The three conserved residues in the sequence motif Px_3_Ix_3_Y (Fig. S1) are more aligned after unwinding of TM5 in opsin (**f**).

Helix parameterization allows us to examine winding or unwinding motions of a helical segment when a protein structure transitions between functional states while the helix preserves its H-bond network in the α conformation. The cytoplasmic segment of TM5 unwinds significantly in opsin, evidenced by a decrease of 3° in angular turn per residue (red dots in top panel of Fig. 2b). However, such unwinding is not observed in the extracellular (or intradiscal) segment of the same helix (red dots in top panel of Fig. 2a). This leads to differential winding as large as 4°/residue between two segments of the same helix. It has caught our attention that a conserved sequence pattern of Px_3_Ix_3_Y in the cytoplasmic segment of TM5 among the opsin family (Fig. S1), in which Pro215, Ile219, and Try223 are located on the same side of this α helix. In rhodopsin, Try223 is exposed on the molecular surface. Unwinding of this helical segment in opsin leads to a better alignment of these three residues, in which all of them face the interior formed by TM3, 5, and 6 (Fig. 2). A new interaction between Arg135^3.50^ [Ballesteros–Weinstein numbering^12^ in superscript] from the conserved sequence motif D(E)RY and Try223 is thus established in opsin^7^,^8^. We further speculate that this unwinding of TM5 in the cytoplasmic segment plays a role in its characteristic elongation upon photoconversion, where Gly224 and Gln225 move their positions to adopt a more regular α helix compared to rhodopsin (Fig. 2e,f).

**Figure 3:**
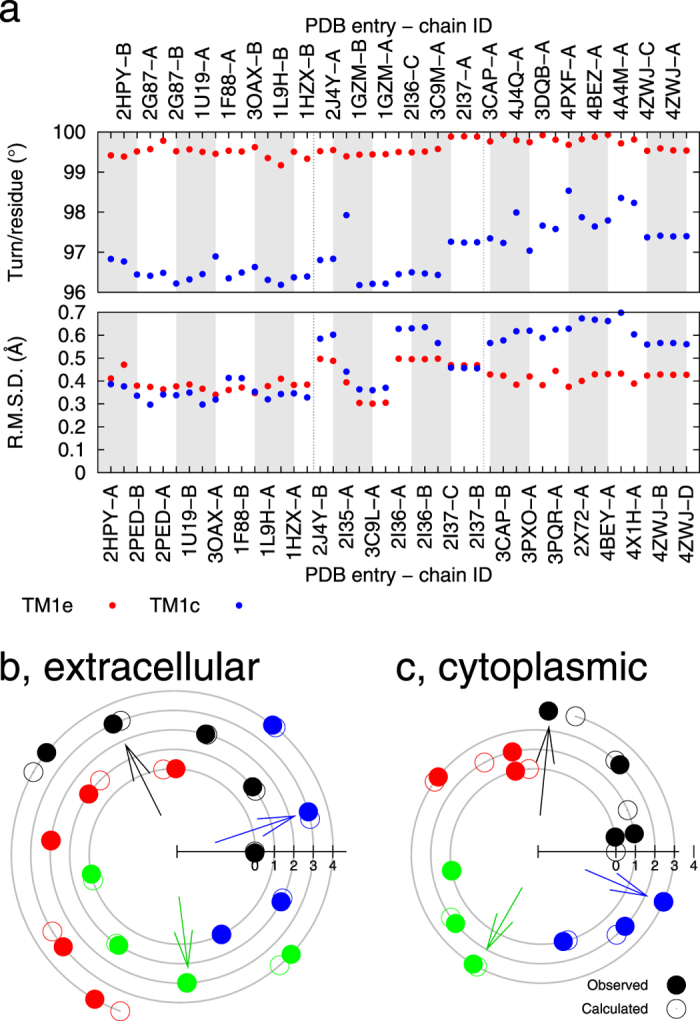
Differential winding of TM1. The angular turn per residue has been refined for both the extracellular and cytoplasmic segments of TM1 (**a**). These segments of the same helix wind to different tightness. The spiral diagrams show the experimentally observed and least-squares fitted phases in dot and circle, respectively, for each residue in the extracellular (**b**) and cytoplasmic (**c**) segments of TM1. The residues located at the N-termini of these segments are placed on the horizontal axes. The three colored arrows mark the 11^th^, 12^th^, and 13^th^ residues counting from the N-terminal residues to show their phase differences resulting from differential winding.

Both segments of TM6 also unwind in opsin but to a lesser extent compared to the cytoplasmic segment of TM5 (blue dots in top panels of Fig. 2a,b). The cytoplasmic segment of TM6 exhibits uniform unwinding like a torsion spring (Movie S1). The phenomenon that two segments of the same helix are wound to different tightness is also observed in TM1 (Fig. 3) and TM7. The extracellular segment of TM1 is consistently overwound relative to the cytoplasmic segment with a greater angular turn of 3°/residue (red dots above blue dots in top panel of Fig. 3a). This difference in angular turn per residue of TM1 shrinks slightly in opsin. Both segments of TM1 remain in good α helix conformation throughout all states. The differential winding of TM7 is discussed below in detail.

Although the angular turn per residue varies by only a few degrees, the cumulative effect can be significant for a long helix (Movie S1). It is plausible that winding/unwinding of helices together with differential winding in helical segments of the same helix facilitate storage of potential energy in various parts of a protein and energy transfer over a long range during the transformation between distinct functional states.

### 3/10, α, and π helices

**Figure 4:**
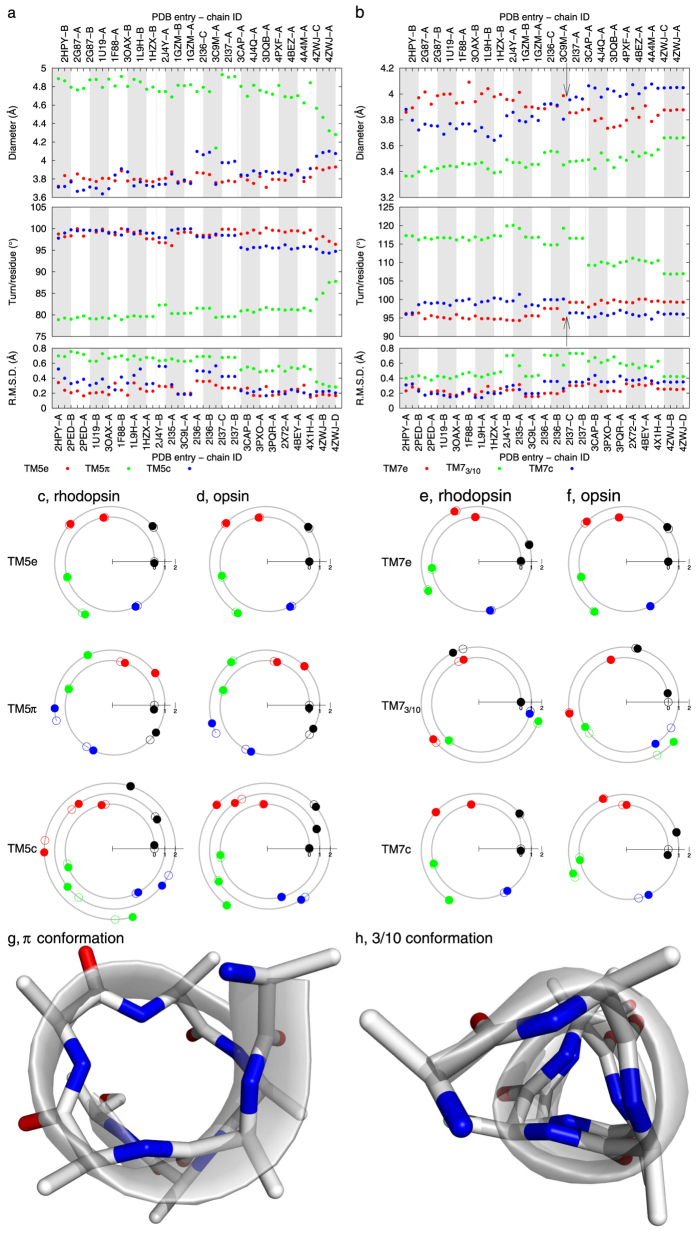
π conformation in TM5 and 3/10 conformation in TM7. The diameter and angular turn per residue of three segments in TM5 and TM7 are plotted in (**a, b**), respectively. Two arrows in (**b**) mark the transition point as the diameter and angular turn of the extracellular and cytoplasmic segments cross over (see main text). Spiral diagrams of three segments of TM5 are compared between rhodopsin (**c**) and opsin (**d**). Three segments of TM7 in rhodopsin (**e**) and opsin (**f**) are also compared. The experimentally observed and least-squares fitted phases of each residue are shown in dot and circle, respectively. The middle segments of TM5 (208–215) and TM7 (293–300) are rendered in ribbon and stick models in (**g, h**), respectively. N and O atoms are in blue and red. Side chains beyond C^β^ are omitted for clarity.

Besides the most common α helix, 3/10 and π helices are also frequently found in membrane proteins (Fig. S1). It is entirely possible that sufficiently large winding and unwinding in a helix eventually triggers transformations between distinct helical forms as seen in another membrane protein sarco/endoplasmic reticulum Ca^2+^-ATPase (Supplementary text and Fig. S5). Although a complete transformation between discrete helical forms has not been captured in rhodopsin, our analysis reveals conformational changes in 3/10 and π helices of rhodopsin. TM5, a relatively straight helix, can be divided into three segments. Both the extracellular and cytoplasmic segments are in α conformation. However, the middle segment (207–215) adopts the π conformation (Fig. S1). As expected, a π helix is ~1 Å wider in diameter than that of a typical α helix (green dots high above red and blue dots in top panel of Fig. 4a) with a decrease of 20°/residue in angular turn (green dots below red and blue dots in second panel of Fig. 4a). As rhodopsin transitions into opsin, the middle π segment of TM5 becomes more tightly wound, evidenced by the changes in both diameter and angular turn per residue, but no transformation to the α conformation occurs. This is likely related to the unwinding of the neighboring cytoplasmic segment that brings Try223 into the molecular interior (see above).

Similarly, TM7 can also be dissected into three segments, where the middle segment (294–301) containing the retinal anchor Lys296 adopts the 3/10 conformation^13^ (Fig. S1). Compared to an α helix, a 3/10 helix exhibits significantly tighter winding with a smaller diameter and a greater angular turn per residue (Fig. 4b). The extent of winding in this anchor helical segment differs by ~5°/residue between rhodopsin and opsin (green dots in second panel of Fig. 4b). Compared to rhodopsin, this middle segment is less regular and barely retains its 3/10 conformation in all known structures determined in the active state (Fig. 4e,f). The flanking extracellular and cytoplasmic segments of TM7 also undergo significant rearrangements. The diameter of the extracellular segment is consistently larger than the cytoplasmic segment by 0.3 Å in the first group of rhodopsin structures (see Methods for grouping of rhodopsin structures). This parameter gradually decreases as rhodopsin transitions to opsin and becomes 0.3 Å less than the cytoplasmic segment (top panel in Fig. 4b), while the angular turn per residue displays the opposite trend (second panel in Fig. 4b). Judging by both diameter and angular turn per residue, these two segments consistent wind differentially and reverse the winding tightness as rhodopsin transitions to opsin (Fig. 4b). In other words, the extracellular and cytoplasmic segments of TM7 adopt the opposite and differential winding states, which alternate between rhodopsin and opsin.

When the cytoplasmic segment of TM7 unwinds in opsin, it inevitably affects the connecting H8 where the conserved sequence motif NPxxYx_5,6_F is located (Fig. S1). In the rhodopsin mutants reconstituted with 9-demethyl retinal analogue^14^, Ala substitutions of the conserved residues in the sequence motif showed that photoconversion to metarhodopsin II is enhanced when one of the aromatic rings in Tyr306 and Phe313 is removed. On the contrary, a disulfide bond bridging between the same two positions impeded photoconversion to metarhodopsin II. The bulky side chains of Tyr306 and Phe313 are in close contact in rhodopsin (Fig. S2), which cannot tolerate the unwinding of TM7 in its cytoplasmic segment (Fig. 4) thus results in a new position of Tyr306 flipped away from Phe313 in opsin^7^,^8^. Lacking the restraint from this close contact would ease the helical unwinding taking place near residue 306 during photoconversion, while a rigid local structure bracketing the corner of TM7 and H8 with a disulfide bridge would hinder the helical unwinding necessary for photoconversion. Taken together, we postulate that TM7 functions as a torsion spring switching between two differentially wound states, and the extended side chain Lys296-retinal located at the middle of the spring acts as a lever (Movie S2). Any changes in the lever and retinal detachment from it alter the winding states of the torsion spring.

More interestingly, this localized structural feature near the anchor site allows us to categorize the structures photoactivated *in crystallo* (related by non-crystallographic symmetry in PDB entry 2I37) into the active opsin group, although their overall structures differ markedly from those of opsin (arrows in Fig. 4b). It is noteworthy that these structures in 2I37 were determined from the yellow crystals obtained by exposing red rhodopsin crystals in ground state to green light^9^,^10^. Such color change indicates that photoconversion is allowed in these photoactive rhodopsin crystals. Our analysis confirms that the retinal anchor helix TM7 indeed undergoes conformational changes similar to those observed in opsin structures. However, the photoconversion *in crystallo* apparently cannot overcome the restraints encountered in this crystal lattice, which prevent the overall reconfiguration of the seven-helical bundle from further transitioning to a completion. As a result, the structures in 2I37 adopt an activated conformation within the immediate vicinity of the chromophore yet situated (perhaps awkwardly as evidenced by the relatively low resolution) in a scaffold largely similar to that of rhodopsin. Although the characteristic color change of the illuminated crystals serves as a direct spectroscopic evidence of photoconversion, it only reflects the conformational changes within the chromophore vicinity. Evidently, these local structural changes permitted by the crystal lattice are decoupled from the rest of the protein such as TM5 and TM6, where large conformational reorganization could only take place without the crystal lattice. Such large reorganization of the seven-helical bundle is silent in absorption spectrum in the visible range. Therefore, photoconversion and activation of rhodopsin are two distinct structural events in its reaction trajectory.

### Torsion and longitudinal sliding

**Figure 5:**
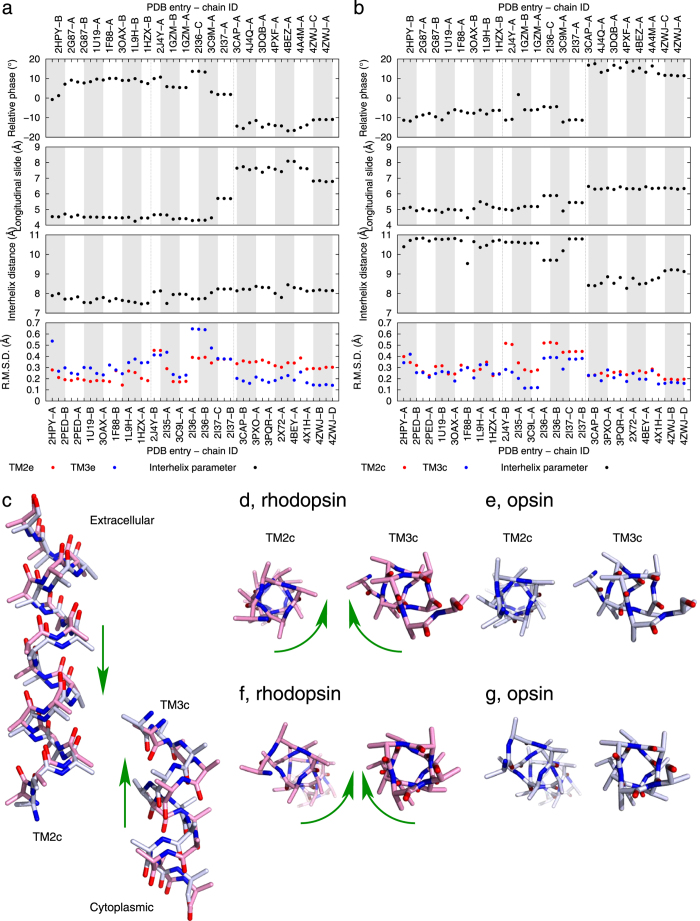
Longitudinal and torsional motions of TM2 and TM3. Relative phase and longitudinal sliding (Methods) between TM2 and TM3 are plotted for their extracellular and cytoplasmic segments in (**a, b**) respectively. The cytoplasmic segments of TM2 and TM3 are rendered in stick models in pink for rhodopsin and in light blue for opsin. N and O atoms are in blue and red. Side chains are omitted except C^β^ for clarity. The straight arrows in (**c**) mark the longitudinal motions from rhodopsin to opsin. (**d, e**) show two states viewed along the cytoplasmic segment of TM2. (**f, g**) are viewed along the cytoplasmic segment of TM3. The curved arrows indicate the torsional motions from rhodopsin to opsin. See also Movie S3 for the relative torque in action.

It is relatively easy to detect scissoring motion and separation of helices based on the crossing angle and distance between two helices when two or more structures are superimposed (Methods). Parameterization of these quantities has been implemented^15^. However, characterization of more complex interhelix relationship requires a more detailed parameterization of helices. Here we examine relative torsional motions between a pair of contacting helices that may occur during the transition from rhodopsin to opsin. Longitudinal, piston-like sliding of a TM helix, also known as axial shift, has been found as a motion component along the helical axis^15^. In rhodopsin, these motions are quite pronounced in roughly parallel TM2 and TM3 in contact on the cytoplasmic side (Fig. 5). These helical segments move both laterally and longitudinally closer to each other in opsin (Fig. 5b,c) and undergo significant torsional motions relative to each other in the meantime (Fig. 5 and Movie S3).

**Figure 6:**
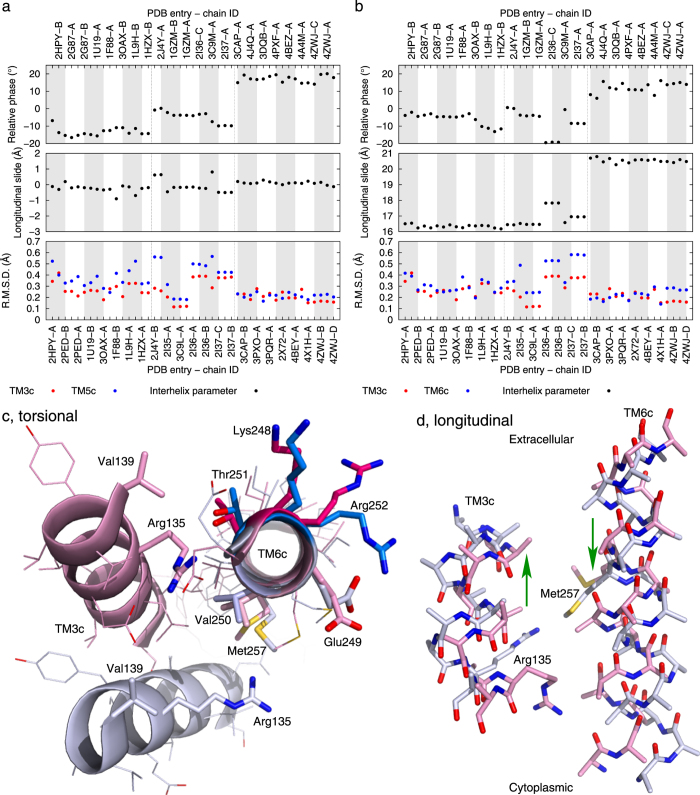
Longitudinal and torsional motions of TM3, 5, and 6. Relative phase changes between the cytoplasmic segments of TM3 and TM5 nicely coincide with the three distinct groups of structures in (**a**) These two segments exhibit no longitudinal sliding. However, the cytoplasmic segments of TM3 and TM6 show both relative phase change and longitudinal sliding in (**b**). These segments are rendered in ribbon and stick models in pink for rhodopsin and in light blue for opsin. N, O, and S atoms are in blue, red, and gold, respectively. The cytoplasmic segment of TM6 is aligned and viewed from the cytoplasmic side in (**c**). Lys248, Thr251, and Arg252 in darker colors on the same side of TM6 are further away from Val139 in opsin than in rhodopsin. Val250 is facing TM3 more directly in opsin. Glu249 maintains its distance to Val139 in both states. The arrows in (**d**) mark the longitudinal sliding of TM3 and TM6 from rhodopsin to opsin, where side chains beyond C^β^ are omitted for clarity except those of Arg135 and Met257.

We identify similar torsion and longitudinal sliding in the cytoplasmic segments of TM3 and TM6 (Fig. 6). The torsional component of TM6 motion was first uncovered by an EPR experiment before the complete rhodopsin structure was determined^11^, which has also been supported by molecular dynamics simulation^16^. The distances between the spin labeled residues in the cytoplasmic segments of TM3 and TM6 increase significantly upon photoactivation. Such outward tilting of TM6 in the active states was later observed in crystallographic structures. Interestingly, the distance between one of the labeled pairs, Val139-Glu249, remained unchanged while a nearby pair, Val139-Val250, appeared to move closer upon activation. These apparent outliers in the EPR observations have been interpreted as a clockwise torsional component of TM6 motion when viewed from the cytoplasmic side^11^. This torsional component is clearly demonstrated in our analysis by the relative phase change in the cytoplasmic segments of TM3 and TM6 (top panel in Fig. 6b) as manifested in the structural superposition based on TM6 alone (Fig. 6c).

C^13^ NMR measurements showed that Arg135 and Met257 are in contact in metarhodopsin I, before a fully developed opsin is formed^17^. The component of longitudinal sliding of TM3 and TM6 has brought them closer (Fig. 6d) while the relative torsional motion facilitates a more direct alignment of them (Fig. 6c). The contact between Arg135 and Met257 in metarhodopsin I suggests that the torsional and longitudinal movements of TM3 and TM6 have occurred before other motions required for metarhodopsin II and opsin states.

**Figure 7:**
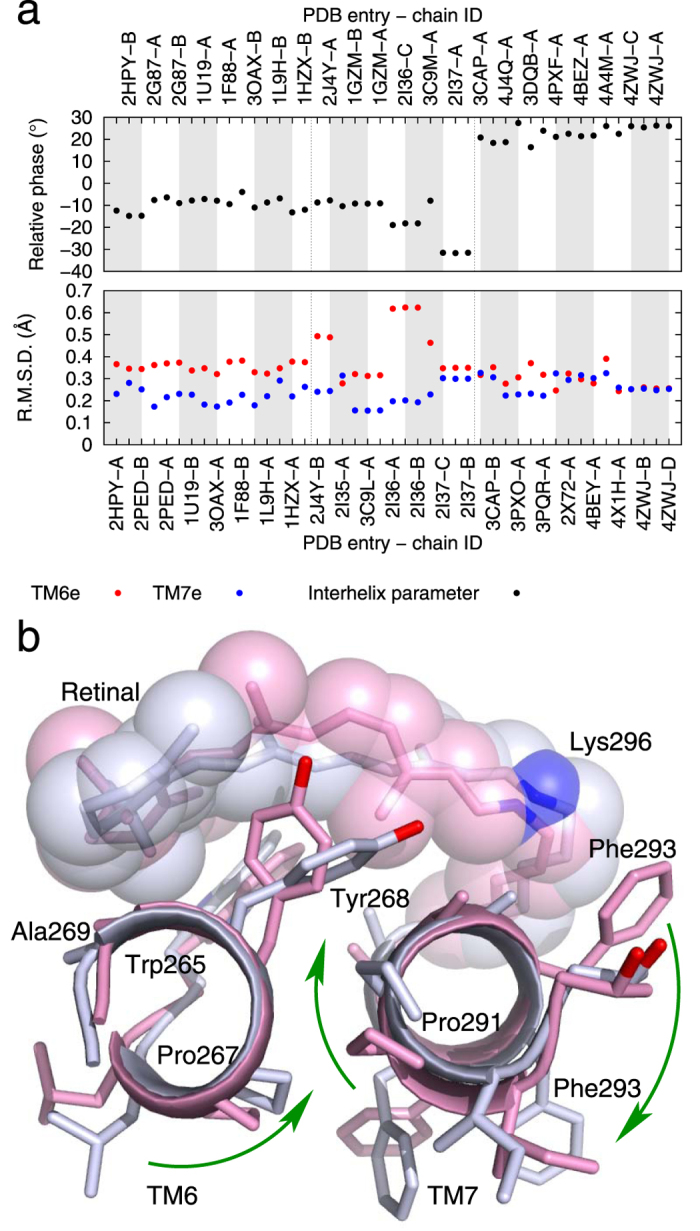
Relative torsion of TM6 and TM7. Significant changes in the relative phase of the extracellular segments of TM6 and TM7 are shown in (**a**). These segments are viewed from the extracellular side in the ribbon and stick models in pink for rhodopsin and light blue for opsin. Retinal and Lys296 are shown in sticks and transparent spheres. N and O atoms are in blue and red, respectively. The curved arrows mark the concerted torsional motions of these segments and the large swing of Phe293. See also Movie S4 for these motions. In TM6, the very conserved Pro267^6.50^ is at a strategically important position next to Tyr268 and Ala269, both of which are in direct contact with the β-ionone ring of the retinal. This Pro residue is also located on the opposite side of Trp265 that forms a large part of the retinal-binding pocket. Pro291 in TM7, conserved among the opsin family (Fig. S1), is about 1.5 turns away from the retinal anchor Lys296. Phe293, on the opposite side from Pro291, swings in concert with the torsional motion of TM7 that opens a small tunnel between TM1 and TM7 to access the retinal binding pocket^7^.

Large relative torsional motions are also found in the extracellular segments of TM6 and TM7 (Fig. 7), from which Pro267 and Pro291 are involved in the solid contacts between the backbones of these helical segments (Fig. S1). Such concerted torsions suggest a motion-coupling mechanism similar to meshing gears^4^ (Movie S4), which may explain how local structural changes induced by photoisomerization of the retinal propagate away and ultimately leading to the formation of the G-protein binding surface. These movements near the chromophore, together with the differentially wound TM7 (Fig. 4), underline the importance of the large relative torsions between the tightly engaged extracellular segments of TM6 and TM7. It is plausible that the large differential winding of TM1, right next to TM7 (Fig. 3), contributes to the activation mechanism. However, the temporal sequence of these structural events is not yet clear, which harbors the cause-effect relationship key to the mechanistic understanding. One approach to quantification of potential intramolecular transmission of force and energy is to perform molecular dynamics simulations under the guidance of the consistently observed structural changes presented here, which is a separate project beyond the scope of this paper.

### Helix compression and stretching

**Figure 8:**
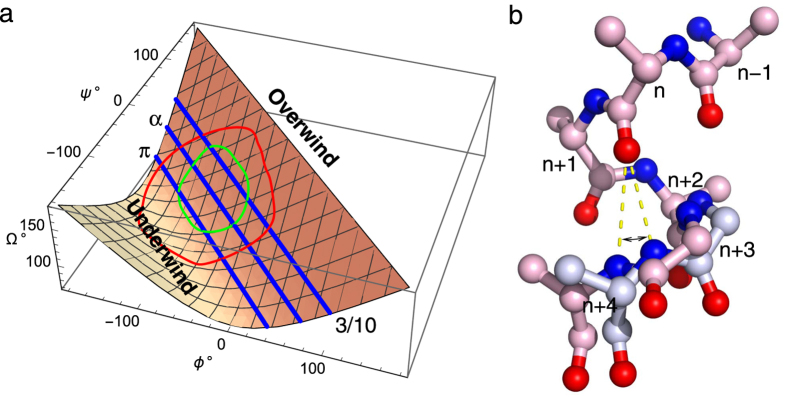
Mechanism of helix winding. (**a**) Relationship between main chain dihedral angles *ϕ, ψ* and angular turn per residue *Ω*. The relationship in Eq. 2 is presented as a meshed surface. The preferred and allowed dihedral angles in helical conformations are circled by the green and red loops, respectively. Three straight lines in blue mark the dihedral angles in ideal conformations of 3/10, α, and π helices, respectively. (**b**) Helix forming main chain H-bond. A short segment of an α helix is rendered as a ball-and-stick model. N and O atoms are in blue and red, respectively. Main chain H-bonds from the carbonyl O of residue *n* to the amide N of residue *n* + 4 are marked as yellow dashed lines. These H-bonds can tolerate a large range in the angle indicated by arrows before the carbonyl O switches partner to residue *n* + 3 or *n*+5 and thus transitions to 3/10 or π helices. This tolerance is the origin of winding and unwinding as the pink and gray structures show. However, the main chain H-bonds tolerate little compression and stretching.

We note that compression and stretching of helices occur less frequently compared to angular motions around the helical axes such as winding, unwinding, and transformation among several helical forms. This is because a main chain H-bond from a carbonyl group to an amide group that forms the helical conformation can be easily angled but less likely compressed and stretched (Fig. 8b). Consequently, we postulate that an intact helix serves as a good mechanical component to transmit pressure, but not tension, over a long distance. Stretching of a helix would lead to its destruction. In contrast, an intact helix has certain capacities to sustain torsion and bending. We show that an angular motion around the helical axis occurs more frequently than previously noticed. The stress due to torsion could be evenly distributed along a long helix as we observe here (Movie S1). We hypothesize that helices in protein structures could serve as torsion springs (Movie S2) to store potential energy and to transmit motion and torque (or turning force). Bending, on the other hand, may lead to stress release through a kink at a location where specific amino acid sequence is embedded in the helix such as Pro and Gly.

## Discussion

In conclusion, our findings suggest that TM helices may transmit force via torsional motions, which are often coupled with helical winding and unwinding. Here we demonstrate that helix parameterization is an effective tool for in-depth analysis of helical conformations that helps dissect complex motion components as a protein structure traverses through a trajectory of different functional states. Forces inferred from these motion components are key to a better understanding of *protein mechanics*^18^. Our analysis of helical conformations enabled by helix parameterization reveals that i) unwinding of TM5 in its cytoplasmic segment is directly responsible for retrieval of Try223 in the sequence motif Px_3_Ix_3_Y from an exposed position on the molecular surface of rhodopsin into the interior of opsin (Fig. 2). ii) The extracellular and cytoplasmic segments of the retinal anchoring TM7 are wound differentially like a torsion spring, in which their winding tightness alternates between different signaling states (Movie S2). Our analysis of the conformational changes in three segments of TM7 explains the structural similarity and distinction between the photoactivated structure *in crystallo* and opsin structures in the active form (Fig. 4b). iii) The torsional component of motion in the cytoplasmic segment of TM6 is isolated from the characteristic outward tilting in opsin, which unambiguously supports the structural interpretation of earlier EPR experiments (Fig. 6). This work provides a comprehensive survey of the conformational dynamics of TM helices embedded in a collection of rhodopsin structures and sheds light on the operating mechanisms of macromolecular systems as electromechanical nano-devices.

## Methods

Structural comparison has been the main tool for detecting structural changes inferred from structural differences among two or more experimentally observed structures. How these structures are aligned as illustrated by molecular graphics software directly influences our interpretation. More than often, this process of discovery heavily relies on visual inspection of the superposed structures. Here we demonstrate that visual comparison of superimposed structures alone is inadequate to detect many components of conformational changes in protein structures. In this work, we devise a simple method of helix parameterization based on least-squares fitting to aid structural comparison.

### Parameterization of helix

The analytical formula to express the main chain conformation of a protein helix is modified^19^. A straight helix can be considered as a superposition of four helices (Fig. 1) of the main chain atoms N, C^α^, C, and O that share the same axis, pitch *s*, and angular turn per residue *Ω*. But each sub-helix of a certain type of main chain atom has a slightly different radius *r*_A_, where A is one of the four main chain atoms. The angular turn per residue is often expressed in its equivalency as number of residues per revolution 2*π*/*Ω*. The Cartesian coordinates of an atom of type A in the straight helix with an orientation or direction cosines ***n*** can be written as:


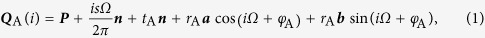


where ***a*** and ***b*** are two orientations normal to each other and both normal to ***n***. They can be obtained by ***a*** = ***n ***× ***c*** ≠ 0 and ***b*** = ***n ***× ***a***, where ***c*** is an arbitrary vector of orientation cosines that is not parallel to ***n***. Since ***a**, **b**, **c***, and ***n*** are vectors of orientation or direction cosines, they all have the same unit length. The axis of the helix passes a reference point ***P*** (Fig. 1). The average of these four radii represents the radius of the overall helix. *t*_A_ and *φ*_A_ represent a small translation along the axis and a phase shift of the sub-helix of atom type A, respectively. Usually, let *t*_C_^α^ = 0 as a reference. *i* is an integer often starting from 0 that represents residue IDs in the helix. A total of 19 parameters plus *t*_C_^α^ fixed at 0 are sufficient to describe all main chain atoms in a straight helix. A bent helix can be treated as two or more straight helices.

Least-squares fitting between the observed atomic coordinates in PDB and a set of calculated coordinates from Eq. 1 provides solutions for all 19 parameters. The location (***P***), orientation (***n***), radius (average of *r*_N_, *r*_C_^α^, *r*_C_, and *r*_O_), pitch (*s*), and angular turn per residue (*Ω*) of a straight helix can be parameterized accurately. Interhelix distance and angle can be calculated subsequently as the distance and angle between two axes. The motion component of the reference point ***P*** along the helical axis is denoted longitudinal sliding. The average of four phase angles for the main chain atoms (*φ*_N_, *φ*_C_^α^, *φ*_C_, and *φ*_O_) indicates the torsional position of a helix. Therefore, its change indicates a torsional motion of the helix. We focus on the relative changes in torsional and longitudinal movements between two helices.

Helix parameterization enables an easy quantification of the intrinsic plasticity of helical conformations using a set of refined geometric parameters. By examining how these parameters vary with the crystal structures determined in different states, we are able to extract subtle yet consistent changes in helical conformations and to study their relevance to protein functions. Thus the significance of a conformational change revealed by helix parameterization is less prone to random noise or systematic factors arising from particular experiments such as underperforming structural refinement. For example, two segments of TM4 exhibit variations in several fitted parameters. Since such variations lack a recognizable trend or bear any correlation with the known functional states of rhodopsin (Fig. S3), they are less likely to be functionally relevant.

The parameters of helical conformation in Eq. 1 are extracted from a set of atomic coordinates by least-squares fitting. As a result, the signals of atomic displacements aggregate in a few intuitive metrics. Although simple structural superposition is often sufficient for identifying large bending and tilting of helices, other conformational changes such as helical winding, torsion, and longitudinal motions are much harder to extract by conventional structural analysis methods. We demonstrate that helix parameterization based on the experimentally determined atomic coordinates allows sensitive and reliable detection of subtle conformational changes.

### Helical winding and main chain dihedrals

The refined dihedral angles of the main chain in protein structures are routinely plotted in Ramachandran diagram for structural validation. These main chain dihedral angles *ϕ* and *ψ* are closely related to the parameter of angular turn per residue *Ω*^20^. Here we rewrite the relationship with minor modifications:


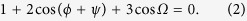


This relationship (Fig. 8a) suggests that the sum of two dihedral angles should be approximately −75°, −105°, and −140°, while the angular turn per residue is 120°, 100°, and 80° in 3/10, α, and π helices, respectively. See Fig. 4 for experimentally observed helical conformations. The continuous distribution of the dihedral angles in the favored areas for 3/10, α, and π helices (Fig. 8a) suggests that these helical conformations are able to undergo smooth interconversion. Even when each helical conformation is maintained, overwinding and unwinding of helices commonly occur in protein structures suggesting a general mechanism for transmitting conformational changes. This work provides a specific example to illustrate that these changes in angular parameters are highly relevant to function, which suggests additional metrics in evaluation of protein structures and new directions for detailed investigation of rhodopsin and other protein structures.

### Plotting of least-squares fitted parameters

Throughout the paper, we present least-squares fitted parameters of various helices (Eq. 1) from all known structures of rhodopsin. These parameters are plotted in multiple panels (for example, Fig. S3). The four-digit alphanumeric code of a PDB entry and the chain ID are listed on the horizontal axis. Vertical dotted lines in each panel divide structures into groups (see below). The value of root mean square deviation (R.M.S.D.) at the convergence of each fitting is plotted in the bottom panel to serve as a quality check. Colored dots are used to distinguish parameters from different helices. Extracellular and cytoplasmic segments are abbreviated as a single letter e or c in labels (for example, TM4e and TM4c). Black dots indicate interhelix parameters such as angle and distance between two helices. The key to each plot is below the plot.

### Spiral diagram

Spiral diagram of the helix, also known as wenxiang diagram^21^, is a concise presentation of helical conformation, especially for the phase positions of all residues in a helix. We adopt spiral diagram to illustrate the helical winding and transformation among various forms of helical conformations. Each residue in a helix is represented by a dot at the averaged position of N, C^α^, C, and O atoms. Usually, the first residue of a helix or a helical segment at its N-terminus starts on a fixed horizontal axis in a spiral diagram. The other residues are arranged on a spiral curve. The residues are colored in cycles of black, red, green, and blue. The experimentally measured positions from PDB are in solid dots; and the calculated positions after least-squares fitting of Eq. 1 are in open circles. Superposition of solid dots and open circles indicates the goodness of fit.

### Rhodopsin structures

PDB has so far accumulated 27 entries of bovine and human rhodopsin structures since the first rhodopsin structure at high resolution was determined^22^. Considering multiple molecules in an asymmetric unit, a total of 45 independently observed structures are currently available. We divide these structures into several groups according to high similarity among a group. The first group includes the ground state rhodopsin structures bound with 11-*cis* retinal together with the early intermediates such as batho and lumirhodopsins. The second group features the elongated TM5 and TM6 at their cytoplasmic ends compared to the first group. Finally, the opsin and metarhodopsin II structures that feature the characteristic tilt of TM6 constitute the last group. These groups are separated by vertical dotted lines in parameter plots.

## Additional Information

**How to cite this article**: Ren, Z. *et al*. Transmembrane Helices Tilt, Bend, Slide, Torque, and Unwind Between Functional States of Rhodopsin. *Sci. Rep.* **6**, 34129; doi: 10.1038/srep34129 (2016).

## Supplementary Material

Supplementary Movie S1

Supplementary Movie S2

Supplementary Movie S3

Supplementary Movie S4

Supplementary Information

## References

[b1] Palczewski K.. G protein–coupled receptor rhodopsin. Annu. Rev. Biochem. 75, 743–767 (2006). 16756510 10.1146/annurev.biochem.75.103004.142743 PMC1560097

[b2] Okada T., Ernst O. P., Palczewski K. & Hofmann K. P.. Activation of rhodopsin: new insights from structural and biochemical studies. Trends Biochem. Sci. 26, 318–324 (2001). 11343925 10.1016/s0968-0004(01)01799-6

[b3] Aravind L. & Ponting C. P.. The cytoplasmic helical linker domain of receptor histidine kinase and methyl-accepting proteins is common to many prokaryotic signalling proteins. FEMS Microbiol. Lett. 176, 111–116 (1999). 10418137 10.1111/j.1574-6968.1999.tb13650.x

[b4] Hulko M. . The HAMP domain structure implies helix rotation in transmembrane signaling. Cell. 126, 929–940 (2006). 16959572 10.1016/j.cell.2006.06.058

[b5] Airola M. V., Watts K. J., Bilwes A. M. & Crane B. R.. Structure of concatenated HAMP domains provides a mechanism for signal transduction. Structure. 18, 436–448 (2010). 20399181 10.1016/j.str.2010.01.013 PMC2892831

[b6] Ferris H. U., Zeth K., Hulko M., Dunin-Horkawicz S. & Lupas A. N.. Axial helix rotation as a mechanism for signal regulation inferred from the crystallographic analysis of the E. coli serine chemoreceptor. J. Struct. Biol. 186, 349–356 (2014). 24680785 10.1016/j.jsb.2014.03.015

[b7] Park J. H., Scheerer P., Hofmann K. P., Choe H.-W. & Ernst O. P.. Crystal structure of the ligand-free G-protein-coupled receptor opsin. Nature. 454, 183–187 (2008). 18563085 10.1038/nature07063

[b8] Scheerer P. . Crystal structure of opsin in its G-protein-interacting conformation. Nature. 455, 497–502 (2008). 18818650 10.1038/nature07330

[b9] Salom D. . Improvements in G protein-coupled receptor purification yield light stable rhodopsin crystals. J. Struct. Biol. 156, 497–504 (2006). 16837211 10.1016/j.jsb.2006.05.003

[b10] Salom D. . Crystal structure of a photoactivated deprotonated intermediate of rhodopsin. Proc. Natl. Acad. Sci. 103, 16123–16128 (2006). 17060607 10.1073/pnas.0608022103 PMC1637547

[b11] Farrens D. L., Altenbach C., Yang K., Hubbell W. L. & Khorana H. G.. Requirement of rigid-body motion of transmembrane helices for light activation of rhodopsin. Science. 274, 768–770 (1996). 8864113 10.1126/science.274.5288.768

[b12] Ballesteros J. A. & Weinstein H.. In Methods in Neurosciences (Elsevier, http://linkinghub.elsevier.com/retrieve/pii/S1043947105800497), vol. 25, pp. 366–428 1995).

[b13] Li J., Edwards P. C., Burghammer M., Villa C. & Schertler G. F. X.. Structure of bovine rhodopsin in a trigonal crystal form. J. Mol. Biol. 343, 1409–1438 (2004). 15491621 10.1016/j.jmb.2004.08.090

[b14] Fritze O. . Role of the conserved NPxxY(x)5,6F motif in the rhodopsin ground state and during activation. Proc. Natl. Acad. Sci. 100, 2290–2295 (2003). 12601165 10.1073/pnas.0435715100 PMC151333

[b15] Dunin-Horkawicz S. & Lupas A. N.. Measuring the conformational space of square four-helical bundles with the program samCC. J. Struct. Biol. 170, 226–235 (2010). 20139000 10.1016/j.jsb.2010.01.023

[b16] Saam J., Tajkhorshid E., Hayashi S. & Schulten K.. Molecular dynamics investigation of primary photoinduced events in the activation of rhodopsin. Biophys. J. 83, 3097–3112 (2002). 12496081 10.1016/S0006-3495(02)75314-9 PMC1302389

[b17] Eilers M. . Structural transitions of transmembrane helix 6 in the formation of metarhodopsin I. J. Phys. Chem. B. 116, 10477–10489 (2012). 22564141 10.1021/jp3019183 PMC3428503

[b18] Bao G.. Protein mechanics: A new frontier in biomechanics. Exp. Mech. 49, 153–164 (2009). 19809588 10.1007/s11340-008-9154-0 PMC2756709

[b19] Ren Z.. Reverse engineering the cooperative machinery of human hemoglobin. PLoS ONE. 8, e77363 (2013). 24312167 10.1371/journal.pone.0077363 PMC3842276

[b20] Hartmann G.. The structure and action of proteins. VonR. E. Dickerson undI. Geis. Harper and Row, Publishers, New York-Evanston-London 1969. 1. Aufl., VIII, 120 S., zahlr. Abb., Paperback DM 20.50. Angew. Chem. 82, 780–780 (1970).

[b21] Chou K.-C., Zhang C.-T. & Maggiora G. M.. Disposition of amphiphilic helices in heteropolar environments. Proteins Struct. Funct. Genet. 28, 99–108 (1997). 9144795

[b22] Palczewski K. . Crystal structure of rhodopsin: A G protein-coupled receptor. Science. 289, 739–745 (2000). 10926528 10.1126/science.289.5480.739

