# Supplementary figures and images for: Transmembrane Helices Tilt, Bend, Slide, Torque, and Unwind between Functional States of Rhodopsin
Source: Sci Rep. 2016 Sep 23;6:34129. doi: 10.1038/srep34129 (PMC5034245; doi:10.1038/srep34129)

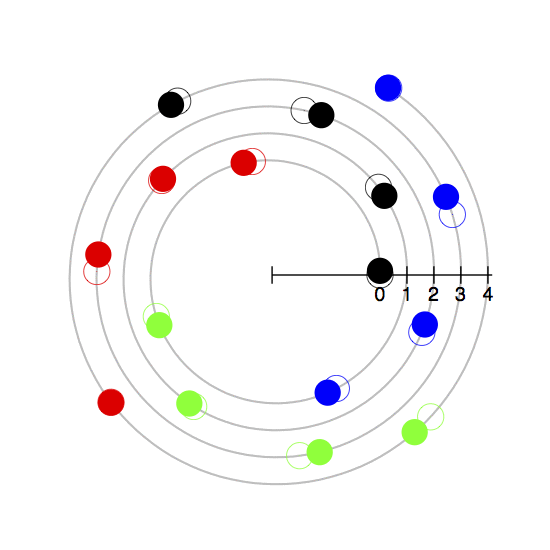

Supplement: Supplementary Movie S1 [file srep34129-s1.gif]

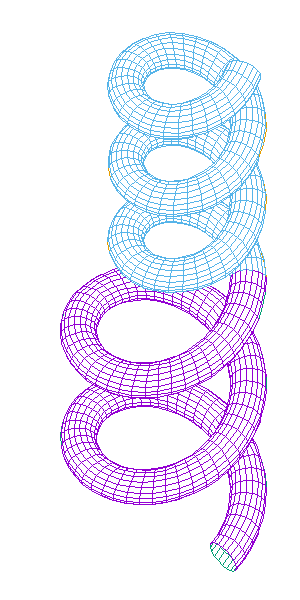

Supplement: Supplementary Movie S2 [file srep34129-s2.gif]

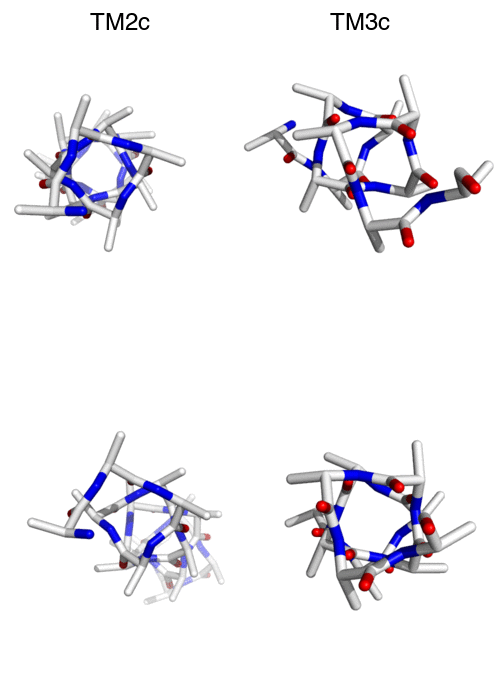

Supplement: Supplementary Movie S3 [file srep34129-s3.gif]

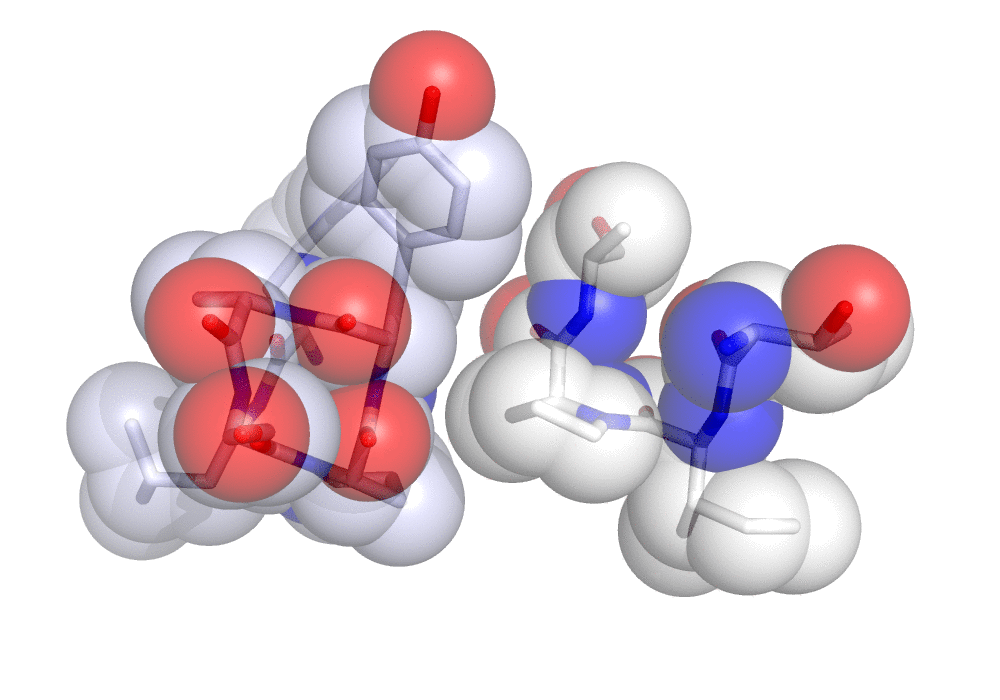

Supplement: Supplementary Movie S4 [file srep34129-s4.gif]
