# Supplementary material for: Transmembrane Helices Tilt, Bend, Slide, Torque, and Unwind between Functional States of Rhodopsin
Source: Sci Rep. 2016 Sep 23;6:34129. doi: 10.1038/srep34129 (PMC5034245; doi:10.1038/srep34129)
Supplement: Supplementary Information [file srep34129-s5.pdf]

Transmembrane Helices Tilt, Bend, Slide, Torque, and Unwind between Functional States of Rhodopsin

Zhong Ren[1,3,5], Peter X. Ren[4,6], Rohith Balusu[4,7], and Xiaojing Yang[1,2,5]

[1]Department of Chemistry, and [2]Department of Ophthalmology and Vision Sciences, University of Illinois at Chicago, Chicago, IL 60607, USA
[3]Renz Research, Inc., Westmont, IL 60559, USA
[4]Hinsdale Central High School, Hinsdale, IL 60521, USA

[5]Corresponding authors: zren@uic.edu and xiaojing@uic.edu
[6]Present address: College of Chemistry, University of California, Berkeley, CA 94720, USA
[7]Present address: University of Illinois at Urbana-Champaign, Urbana, IL 61801, USA

Supplementary Information

Helix bending

Large helix bending is readily detectable by superposition of multiple structures. Finding a subtle bend, however, benefits from computational tools such as a crease finding algorithm (*18*). Even a slight bend could be an indication of functional motions. One such example is from a dimeric hemoglobin structure of invertebrates, in which a small but consistent bend at the middle of helix E was identified as a key evidence to support the mechanical coupling in cooperative oxygen binding (*18*).

The seven transmembrane (TM) helices of rhodopsin exhibit various extents of bending among numerous structures determined thus far (*13*, *19*). TM2 displays the most significant change in bending with an angle as large as 30° in rhodopsin, which decreases to about 10° – 15° in opsin (Fig. S4a). Between the two straight segments in α helical conformation, a few residues (86-89) experiencing the bending bulge out in a π helical conformation or lose the helical conformation completely. As a result, TM2 bends rather freely at this location without interrupting the contiguous helical segments on both sides (Fig. S4c).

Unlike TM2, TM6 retains its severe bend around 36° throughout all states of rhodopsin/opsin (Fig. S4b). This persistent bend is caused by $Pro267^{6.50}$ at the junction of the two straight segments. The large conformational changes of TM5 and TM6 in rhodopsin and other GPCRs have been directly attributed to

formation and destruction of the G-protein binding surfaces. Indeed, our calculations show that the interhelix angle between the cytoplasmic segments of TM5 and TM6 changes by an average of 25° from rhodopsin to opsin structures (third panel in Fig. 2b). These helices become nearly parallel and much closer to each other in opsin (*7*, *8*). Surprisingly, these large changes do not seem to affect the bending angle of TM6 (Fig. S4b), in support of the rigid body seesaw movement (*20*, *21*). However, the cytoplasmic segment of TM6 undergoes consistent unwinding in opsin (Fig. 2b and Movie S1; see main text).

Helix transformation

Several helical forms 3/10, α, and π provide relatively stable energy minima for a protein structure in helical conformations. Complete transformation among these conformations has not been observed in rhodopsin. However, transformation is possible in TM helices as shown in sarco/endoplasmic reticulum Ca^{2+}-ATPase (SERCA). SERCA is a member from P-type ATPases that pumps Ca^{2+} into sarcoplasmic reticulum during muscle relaxation as driven by ATP hydrolysis (*22*).

M6, a relatively short TM helix in SERCA, exhibits two clearly discontinued segments due to Pro803 that breaks the helical H-bond network. Its cytoplasmic segment transforms to the 3/10 conformation in E1 state from α form in E2 state (Fig. S5). This relatively long 3/10 helix in E1 state spans from 801 to 810 with one glitch at the carbonyl of Pro803 in its main chain H-bonds. However, the same segment in E2 state becomes α conformation except that the last turn remains in the 3/10 conformation.

Transformation between 3/10 and α conformations occurs in the C-terminal segment of M3 in SERCA as well (275-282). This short α helix in E1 state frequently becomes a 3/10 helix in E2 state. However, this transition is less consistent in the available structures of SERCA.

Supplementary Figures and Legends

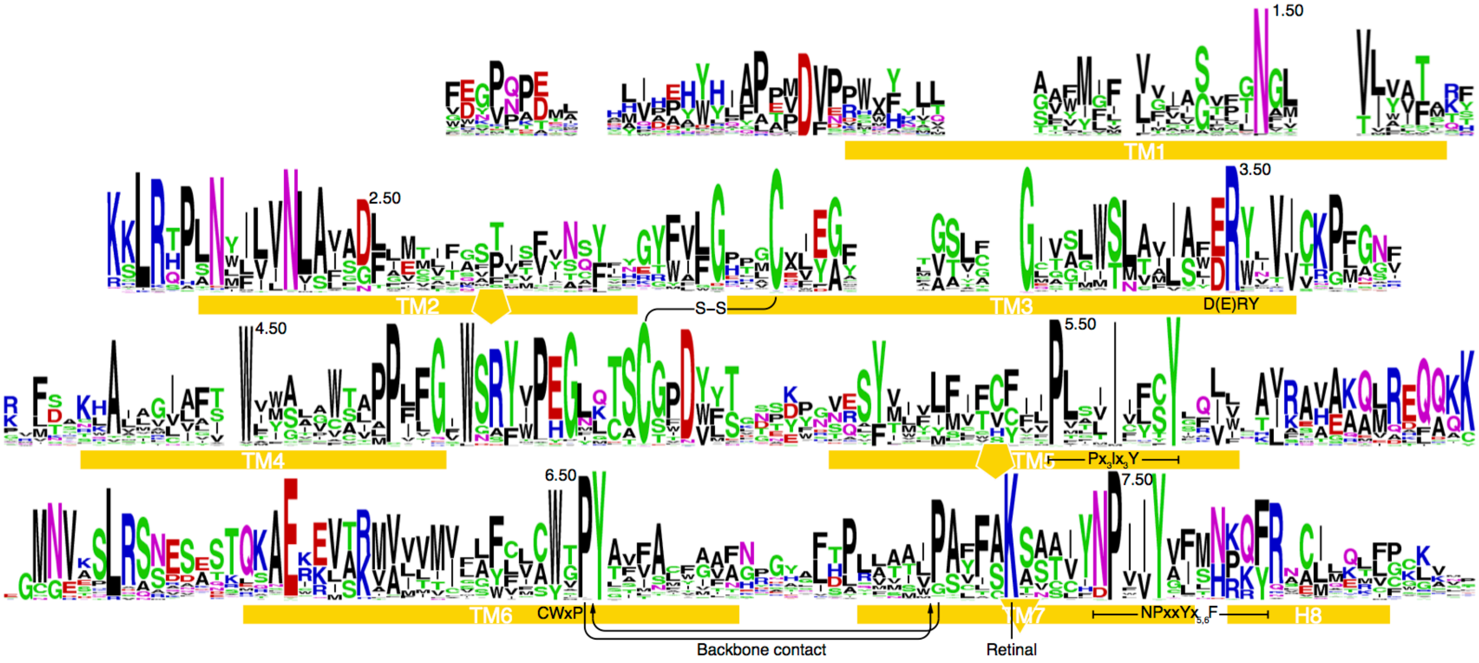

Figure S1. Protein sequence alignment in opsin family. The height of each character corresponds to the amino acid conservativeness. The yellow bars underline TM1-7 and H8. The yellow triangle and pentagons mark the locations of the segments in 3/10 and π conformations, respectively. Several sequence motifs and other structural features are annotated.

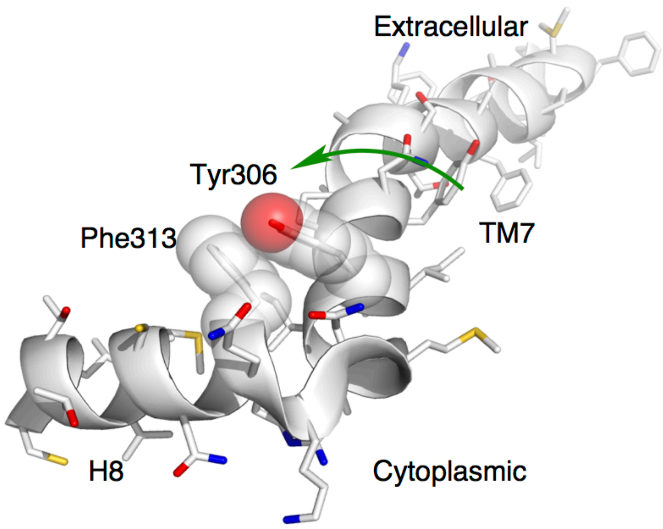

Figure S2. TM7-H8 corner. The structure of TM7-H8 is rendered as ribbon and stick model. N, O, and S atoms are in blue, red, and gold, respectively. Tyr306 and Phe313 in spheres from the conserved sequence motif NPxxYx$_{5,6}$F are in close contact in rhodopsin. The green arrow marks the direction of unwinding as rhodopsin transitions to opsin (Fig. 4).

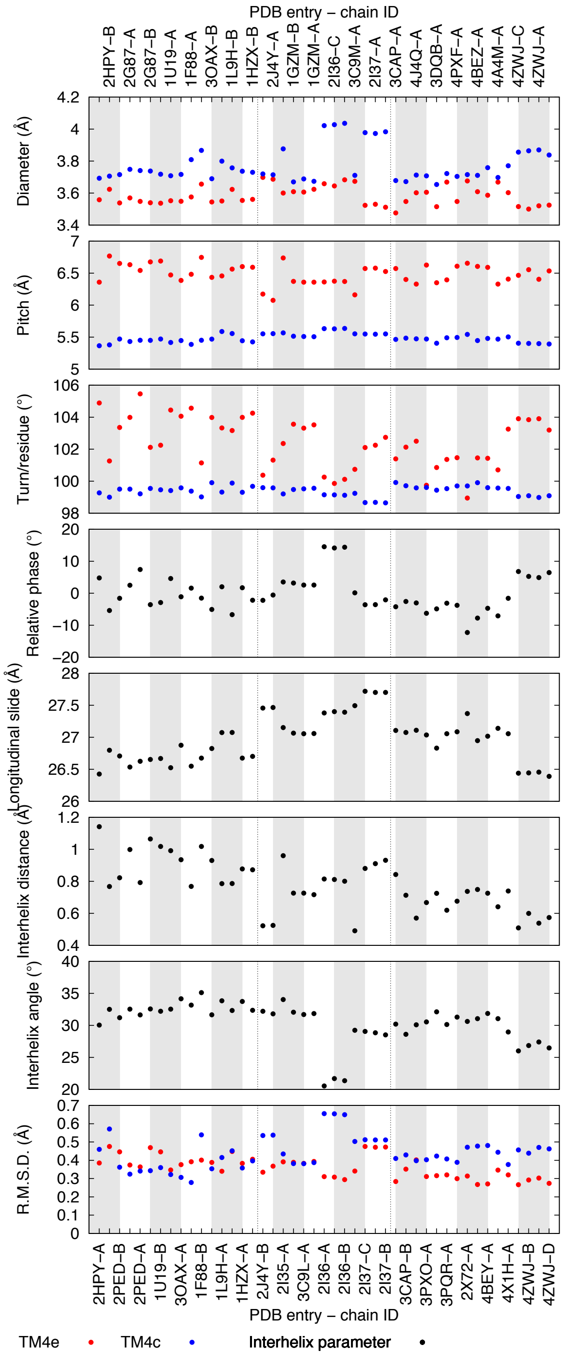

Figure S3. Refined parameters of TM4. Two consecutive Pro residues at 170 and 171 introduce a kink in TM4, thus divide this helix into extracellular and cytoplasmic segments. Diameter, pitch, and angular turn per residue of these segments are fitted to all available structures of bovine and human rhodopsins. The extracellular segment is in a hybrid conformation somewhere between α and 3/10 and is too short to define its parameters accurately. Therefore, the parameters of the extracellular segment in red appear to scatter more widely

than those of the cytoplasmic segment in blue. The rmsd values of the fitting are plotted in the bottom panel. The parameters in black that describe the geometric relationship of two helical segments are also calculated, such as relative phase, longitudinal sliding, interhelix distance, and angle.

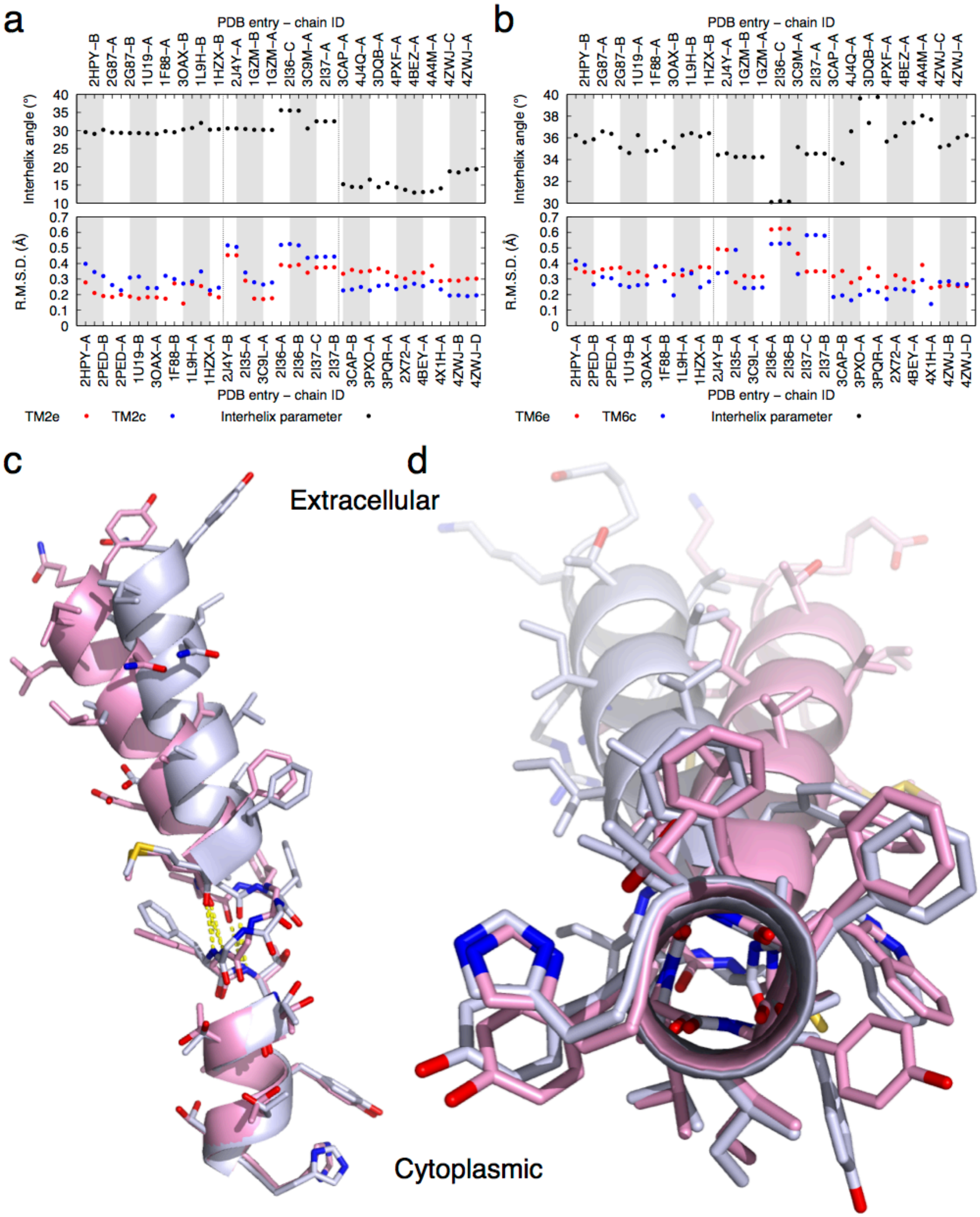

Figure S4. Helix bending of TM2 and TM6. The calculated bending angles between the extracellular and cytoplasmic segments are plotted in the top panels of a and b. The differences in bending of TM2 and TM6 are shown in c and d, respectively, when the cytoplasmic segments are aligned. Rhodopsin is in pink and opsin is in light blue. N, O, and S atoms are in blue, red, and gold, respectively. TM2 is displayed in the plane of the bending angle (c). It is less

bent in opsin than in rhodopsin. H-bonds Met86O-Phe91N and Val87O-Thr92N that form the π conformation are depicted as yellow dashed lines. TM6 is viewed along the cytoplasmic segment (d). The bending angle of TM6 fluctuates around 36°±5° in all structures without a consistent trend. However, the extracellular segment swings around the axis of the cytoplasmic segment. This swing is consistent with the unwinding motion of the cytoplasmic segment of TM6 in opsin. See this motion of unwinding in Movie S1.

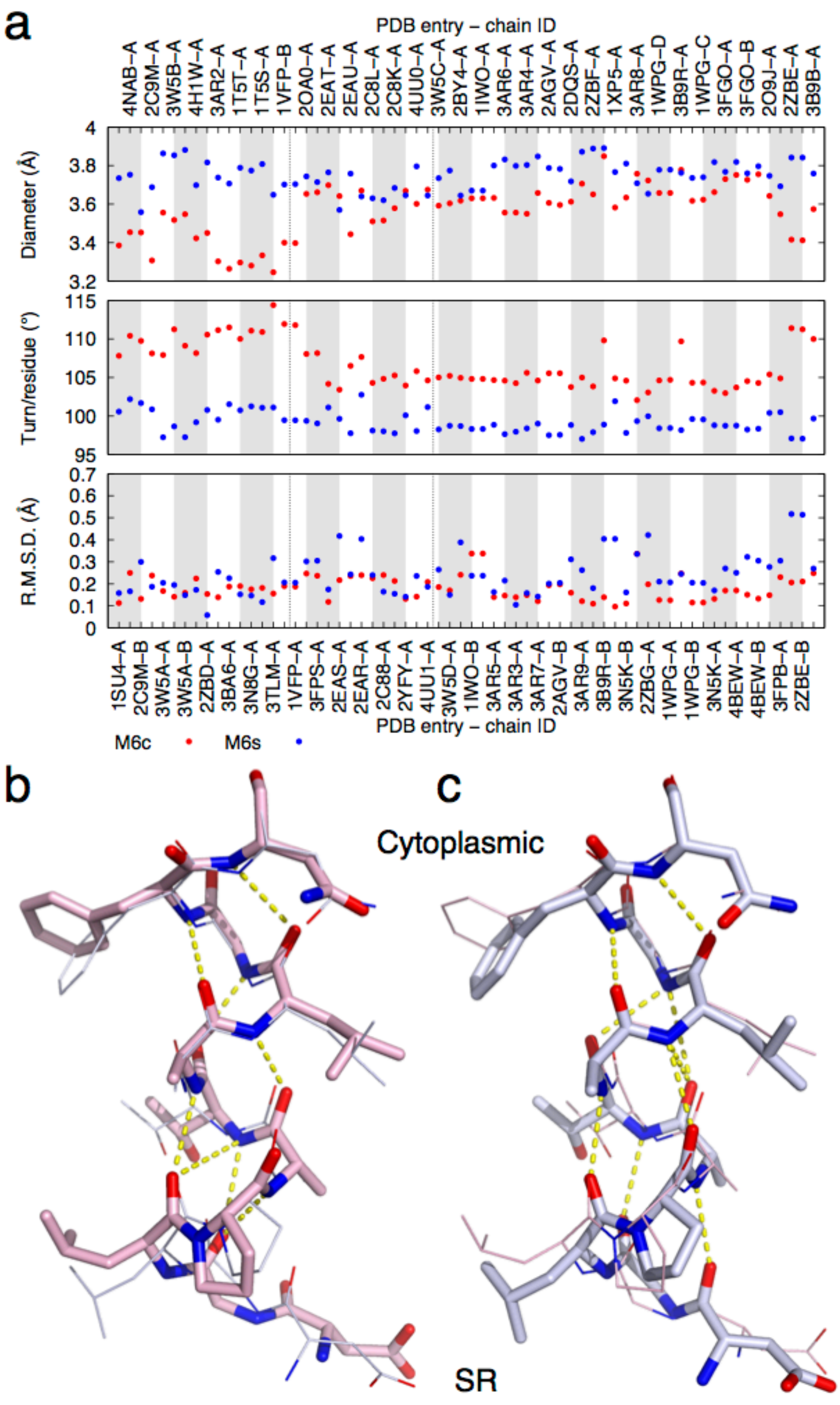
a
PDB entry – chain ID
4NAB–A 2C9M–A 3W5B–A 4H1W–A 3AR2–A 1T5T–A 1T5S–A 1VFP–B 2OA0–A 2EAT–A 2EAU–A 2C8L–A 2C8K–A 4UU0–A 3W5C–A 2BY4–A 1IWO–A 3AR6–A 3AR4–A 2AGV–A 2DQS–A 2ZBF–A 1XP5–A 3AR8–A 1WPG–D 3B9R–A 1WPG–C 3FGO–A 3FGO–B 2O9J–A 2ZBE–A 3B9B–A
Diameter (Å)
4 3.8 3.6 3.4 3.2
Turn/residue (°)
115 110 105 100 95
R.M.S.D. (Å)
0.7 0.6 0.5 0.4 0.3 0.2 0.1 0
1SU4–A 2C9M–B 3W5A–A 3W5A–B 2ZBD–A 3BA6–A 3N8G–A 3TLM–A 1VFP–A 3FPS–A 2EAS–A 2EAR–A 2C88–A 2YFY–A 4UU1–A 3W5D–A 1IWO–B 3AR5–A 3AR3–A 3AR7–A 2AGV–B 3AR9–A 3B9R–B 3N5K–B 2ZBG–A 1WPG–A 1WPG–B 3N5K–A 4BEW–A 4BEW–B 3FPB–A 2ZBE–B
PDB entry – chain ID
M6c
M6s
b
c
Cytoplasmic
SR

Figure S5. Cytoplasmic segment of M6 in SERCA. Diameter and angular turn per residue of the cytoplasmic segment of M6 are determined for 64 structures of SERCA in PDB. The structures in E1 state are arranged at the beginning of the list. The cytoplasmic segment in red shows smaller diameter and greater turn per residue (a). This segment is rendered in stick models in b and c. E1 and E2 states are in pink and light blue, respectively. N and O atoms are in blue and red. Main chain H-bonds that form the helix are depicted in yellow dashed lines. The thicker pink model in E1 state features H-bonds of 3/10 conformation (b). The thicker blue model in E2 state is in α conformation except the last turn at its C-terminus remained in 3/10 conformation (c).

Supplementary Movies

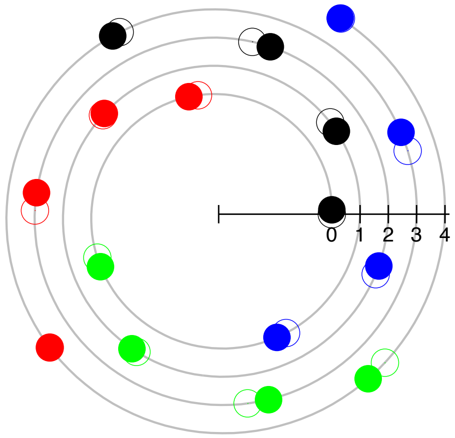

Movie S1. Spiral diagrams of the cytoplasmic segment of TM6. This movie flashes between two spiral diagrams (Methods) between rhodopsin and opsin. The cytoplasmic segment of TM6 exhibits significant and uniform unwinding along its entire length when the protein transitions to opsin. All residues in this segment move consistently towards the same direction, which resembles the motion of a torsion spring. This unwinding is correlated with a motion that the extracellular segment of the same helix swings in the counterclockwise direction around the axis of this torsion spring when viewed from the cytoplasmic side (Fig. S4bd). However, it is uncertain which motion is the cause or consequence of the other.

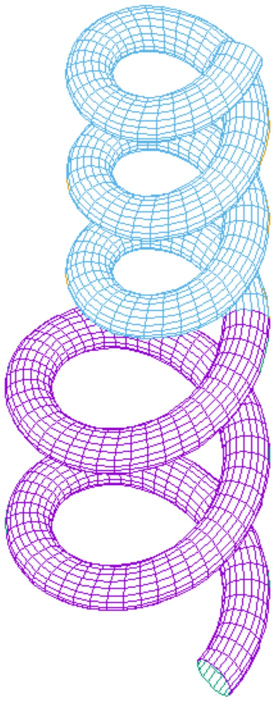

Movie S2. Simulation of torsion spring. This simulation shows that a torsion spring is acted on at the middle intersection where two segments in different colors join together. One segment is overwound while the other is unwound. The diameter and tightness of winding switch back and forth. These motions are exaggerated compared to those found in TM7 (Fig. 4).

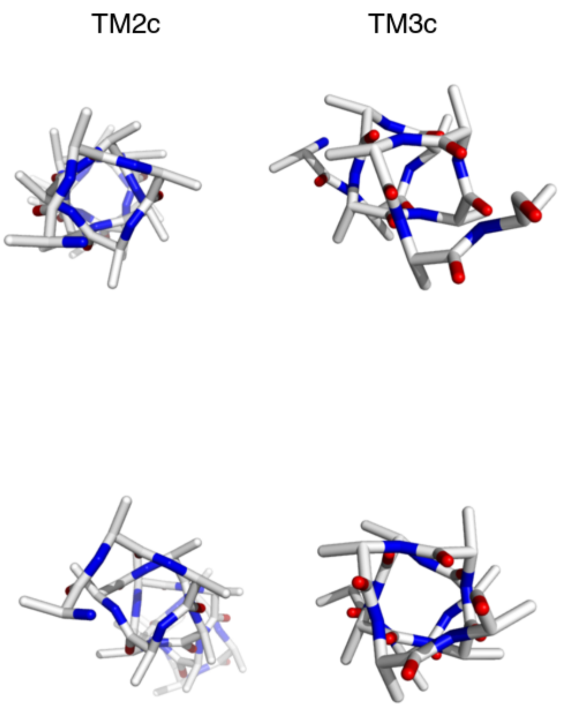

Movie S3. Relative torsional motions between the cytoplasmic segments of TM2 and TM3. The top and bottom rows are viewed along the axes of the cytoplasmic segments of TM2 and TM3, respectively. See Fig. 5 for more detail.

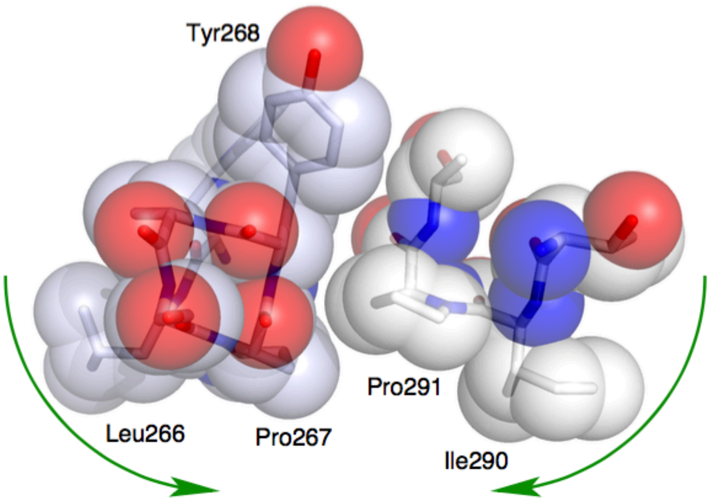

Movie S4. Relative torsional motions between the extracellular segments of TM6 and TM7. The extracellular segments of TM6 in light blue and TM7 in white near the engaged Pro267$^{6.50}$ and Pro291 are viewed from the extracellular side. N and O atoms are in blue and red, respectively. The arrows mark the torsional motions from rhodopsin to opsin. See Fig. 4 for more detail.
